# Not only synovitis but also tenosynovitis needs to be considered: why it is time to update textbook images of rheumatoid arthritis

**DOI:** 10.1136/annrheumdis-2019-216350

**Published:** 2019-12-19

**Authors:** Cleo Rogier, Silvia Hayer, Annette van der Helm-van Mil

**Affiliations:** 1 Rheumatology, Erasmus Medical Center, Rotterdam, Zuid-Holland, The Netherlands; 2 Division of Rheumatology, Medical University of Vienna, Vienna, Austria; 3 Rheumatology, Leiden University Medical Center, Leiden, The Netherlands

**Keywords:** rheumatoid arthritis, magnetic resonance imaging, synovitis

Rheumatoid arthritis (RA) is typically represented as synovitis and bone erosions of small joints. This classic picture resulted from comparing patients with RA with other rheumatic joint diseases for clinical and radiographic characteristics. Although different classification criteria for RA have been developed over time, this classic picture has not changed since the mid-20th century. During the last decennium, advanced imaging modalities, such as MRI and musculoskeletal ultrasound (US), have refined our understanding of tissues involved in RA. We will argue that tenosynovitis at the level of the hand and feet joints is a feature that deserves to be added as the third classic trait of RA.

A feature can be considered as a disease trait when it occurs frequently and is specific, and when a new trait is considered its connection with the disease is not a substitute of an already acknowledged classic feature. We will study the occurrence of tenosynovitis in RA in the light of these principles.

Many, but not all, tendons at the hand and feet joints are surrounded by a sheath.^1 2^ Tendon sheaths have a cell composition similar to the synovial lining of joints.^3^

**Figure 1:**
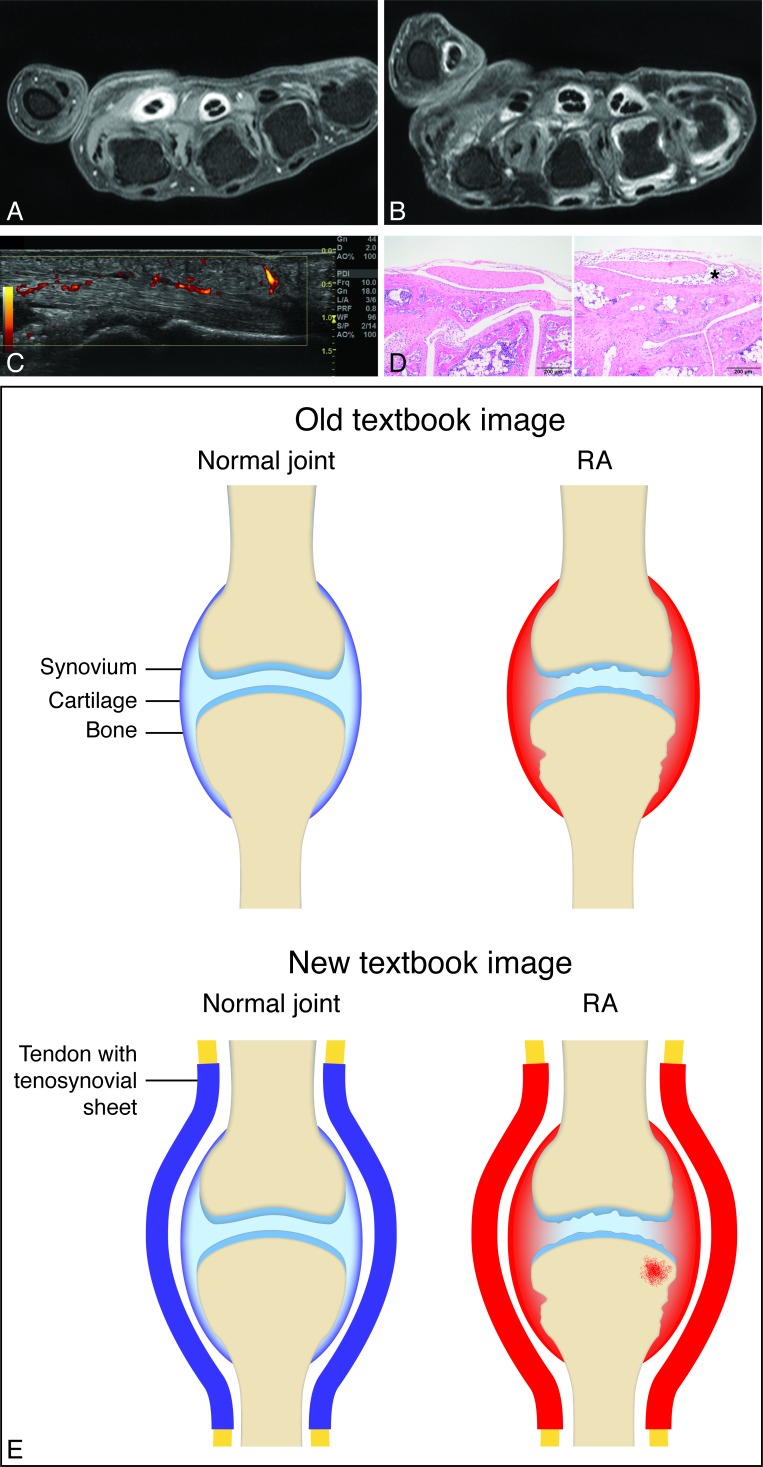
Tenosynovitis as an early trait in RA (A–C) and experimental arthritis (D), and the proposed new textbook image of RA including tenosynovitis (E). (A–B) MRI (axial plane after contrast enhancement, 1.5T MRI) of two patients with early RA with flexor tenosynovitis at MCP 2 and 3 (A) and flexor tenosynovitis at MCP 1, 3 and 4, and synovitis at MCP 4 and 5 (B). (C) Ultrasound (longitudinal plane) in a patient with early RA showing flexor tenosynovitis at MCP 2. (D) H&E–stained transverse section planes of the hind paw of 4-week-old wild-type (left) and hTNFtg (right) arthritis mice with tenosynovitis (*infiltration of lymphocytes and inflammation of the tendon sheath) in the preclinical phase of arthritis (magnification 100×). (E) Proposed new textbook image with tenosynovitis and osteitis. MCP, metacarpophalangeal; RA, rheumatoid arthritis.

Fiona McQueen was the first to describe tenosynovitis in early RA using MRI.^4^ The reported prevalence of tenosynovitis depends on the number of tendon sheaths studied (wrist, metacarpophalangeal (MCP) and/or metatarsophalangeal (MTP) joints, unilateral or bilateral). A prevalence of ~50% is described,^5 6^ but most were higher (~80%).^7–11^ MRI studies in consecutive early RA showed a sensitivity of tenosynovitis of 75%–87%.^7–9^ Figure 1A–C presents imaging examples (MRI, US) in early RA. Imaging studies in persons from the general population repetitively showed a prevalence of tenosynovitis at small joints ranging from 0% to 3%,^12–14^ corresponding with a specificity of 97%–100%. The specificity in patients with other arthritides as reference is also high. A study at the tendon level of the wrist and MCP joints, comparing consecutive patients with RA and other early arthritis (including psoriatic arthritis), reported a specificity ranging from 82% to 99%.^8^ Thus, tenosynovitis at the level of small joints (MCPs, wrist, MTPs) has high sensitivity and specificity for RA.

Studies in an experimental mouse model showed that tenosynovitis was the first sign of inflammation.^15^ Infiltration of the tendon sheaths by granulocytes and macrophages was the first pathological event in the preclinical phase; only few T cells were present and B cells were initially absent (figure 1D). Hyperplasia of the joint synovial lining was observed at the onset of clinical arthritis but not in the preclinical disease.^15^ The question if tenosynovitis is also the initiating feature of arthritis in humans with RA is still unsolved. However, a serial MRI study in pre-RA revealed that tenosynovitis and synovitis occurred very early, before the development of clinical arthritis and erosions.^16^ The notion that tenosynovitis is a very early feature of RA is further supported by the consistent finding that tenosynovitis is an independent predictor for developing RA in patients with clinically suspect arthralgia and undifferentiated arthritis, whereas synovitis is not constantly predictive in multivariate analysis (online supplementary table).

10.1136/annrheumdis-2019-216350.supp1 Supplementary data

Finally we explored whether tenosynovitis contributes to symptoms and signs that are characteristic of RA. A summary of currently available data reveals that tenosynovitis is related to the presence of joint swelling, joint tenderness, morning stiffness and functional impairments in RA and in earlier disease phases (online supplementary table). Associations were independent of possible concomitant imaging-detected synovitis.

To summarise, tenosynovitis at the level of small joints has high sensitivity and specificity for early RA. Tenosynovitis occurs early during RA development. It underlies symptoms and signs that are characteristic of RA, both in preclinical stages and in clinical RA. Based on this we propose that, in addition to synovitis and structural damage, future textbook images from now on also depict tenosynovitis as a classic trait of RA, as portrayed in figure 1E. In addition, if classification criteria for the earliest phases of RA were to be derived or modified, tenosynovitis could be included.
