# Supplementary material for: Not only synovitis but also tenosynovitis needs to be considered: why it is time to update textbook images of rheumatoid arthritis
Source: Ann Rheum Dis. 2019 Dec 19;79(4):546–7. doi: 10.1136/annrheumdis-2019-216350 (PMC7147173; doi:10.1136/annrheumdis-2019-216350)
Supplement: Supplementary data [file annrheumdis-2019-216350supp001.pdf]

Supplementary Table. Association of imaging detected tenosynovitis at small joints with progression from pre-RA stages to RA, as well as with symptoms and signs that are characteristic for RA. Shown are the results from univariable analyses, and multivariable analyses that adjusted for concomitant presence of imaging detected synovitis, performed in two phases of RA development and in RA.

Clinically Suspect Arthralgia | **Undifferentiated/Early Arthritis** | **Rheumatoid Arthritis**

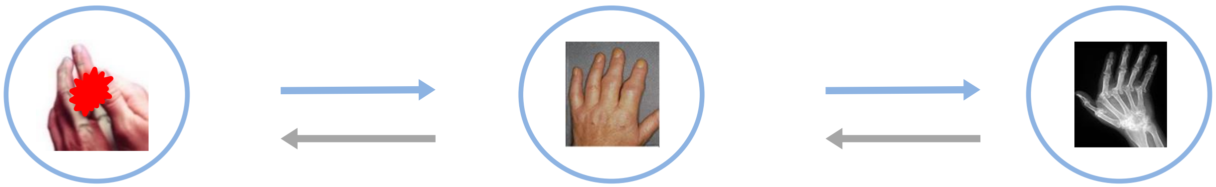

	Tenosynovitis		Synovitis			Tenosynovitis		Synovitis			Tenosynovitis		Synovitis	
	Uni	*Multi*	*Uni*	*Multi*		*Uni*	*Multi*	*Uni*	*Multi*		*Uni*	*Multi*	*Uni*	*Multi*
**Predicting RA**														
Kleyer (1)	✓	NR	NR	NR	Eshed (5)	✓	✓	✓	x		NA	NA	NA	NA
Steenbergen (2) ¥	✓	✓	✓	x	Navalho (6)	✓	✓	✓	✓					
Matthijssen (3) ¥	NR	✓	NR	x	Nieuwenhuis (7)	✓	✓	✓	x					
Hensvold (4)	✓	NR	NR	NR	Sahbudin (8)	✓	✓	✓	✓					
					Dakkak (9)	✓	✓	x	x					
**Joint Swelling**														
					Krabben (10)	✓	✓	✓	✓	Krabben (10) §	✓	✓	✓	✓
**Tenderness, pain**														
Burgers (11)	✓	x	✓	✓	Krabben (10)	✓	✓	✓	✓	Krabben (10) §	✓	✓	✓	✓
										Mo (12)	NR	✓	NR	✓
**Morning stiffness**														
					Boer (13) ∞	✓	NR	✓	NR	Kobayashi (14)	✓	✓	✓	x
										Nishino (15)	✓	NR	x	NR
										Boer (13) ∞ §	✓	NR	✓	NR
**Functional impairment**														
Ten Brinck (16)	✓	NR	x	NR	Burgers (17)	✓	✓	✓	x	Burgers (17) §	✓	✓	✓	x
										Nishino (15)	x	NR	✓	NR
										Glinatsi (18)	✓	✓	✓	x

NA: not applicable, NR: not reported

Uni: results of univariable analysis, Multi: results of multivariable analysis including tenosynovitis and synovitis

✓: associated, X: not associated (not statistically significant)

∞ stratified analysis

¥ partly same cohort

§ sub-analysis of patients fulfilling 2010 ACR/EULAR criteria for Rheumatoid Arthritis.

*Numbers used in table are referring to supplementary references

SUPPLEMENTARY REFERENCES

1 Kleyer A, Krieter M, Oliveira I, et al. High prevalence of tenosynovial inflammation before onset of rheumatoid arthritis and its link to progression to ra-a combined mri/ct study. *Semin Arthritis Rheum* 2016;46:143-150.

2 van Steenbergen HW, Mangnus L, Reijnierse M, et al. Clinical factors, anticitrullinated peptide antibodies and mri-detected subclinical inflammation in relation to progression from clinically suspect arthralgia to arthritis. *Ann Rheum Dis* 2016;75:1824-1830.

3 Matthijssen X, Wouters F, Boeters D, et al. Improving and validating the predictive accuracy of mri detected subclinical inflammation for rheumatoid arthritis development in clinically suspect arthralgia [abstract]. *Ann Rheum Dis* 2019;78(Suppl 2):P1024.

4 Hensvold A, Kisten Y, Circiumaru A, et al. Development of ultrasound detectable arthritis among acpa positive subjects with musculoskeletal symptoms: The risk ra prospective study [abstract]. *Ann Rheum Dis* 2019;78(Suppl 2):P310

5 Eshed I, Feist E, Althoff CE, et al. Tenosynovitis of the flexor tendons of the hand detected by mri: An early indicator of rheumatoid arthritis. *Rheumatology (Oxford)* 2009;48:887-891.

6 Navalho M, Resende C, Rodrigues AM, et al. Bilateral evaluation of the hand and wrist in untreated early inflammatory arthritis: A comparative study of ultrasonography and magnetic resonance imaging. *J Rheumatol* 2013;40:1282-1292.

7 Nieuwenhuis WP, van Steenbergen HW, Mangnus L, et al. Evaluation of the diagnostic accuracy of hand and foot mri for early rheumatoid arthritis. *Rheumatology (Oxford)* 2017;56:1367-1377.

8 Sahbudin I, Pickup L, Nightingale P, et al. The role of ultrasound-defined tenosynovitis and synovitis in the prediction of rheumatoid arthritis development. *Rheumatology (Oxford)* 2018;

9 Dakkak YJ, Boeters DM, Boer AC, et al. What is the additional value of mri of the foot to the hand in undifferentiated arthritis to predict rheumatoid arthritis development? *Arthritis Res Ther* 2019;21:56.

10 Krabben A, Stomp W, Huizinga TW, et al. Concordance between inflammation at physical examination and on mri in patients with early arthritis. *Ann Rheum Dis* 2015;74:506-512.

11 Burgers LE, Ten Brinck RM, van der Helm-van Mil AHM. Is joint pain in patients with arthralgia suspicious for progression to rheumatoid arthritis explained by subclinical inflammation? A cross-sectional mri study. *Rheumatology (Oxford)* 2019;58:86-93.

12 Mo Y, Yang Z-H, Wang J-W, et al. Mri-detected digit flexor tenosynovitis in bilateral proximal interphalangeal joints contribute to joint tenderness in patients with early rheumatoid arthritis. [abstract]. *Ann Rheum Dis* 2019;78:(Suppl 2):P1008.

13 Boer A, Boeters D, Niemantsverdriet E. The contribution of tenosynovitis of small joints to the symptom morning stiffness in patients presenting with undifferentiated and rheumatoid arthritis [abstract]. *Ann Rheum Dis* 2019;78(Suppl 2):P692

14 Kobayashi Y, Ikeda K, Nakamura T, et al. Severity and diurnal improvement of morning stiffness independently associate with tenosynovitis in patients with rheumatoid arthritis. *PLoS One* 2016;11:e0166616.

15 Nishino A, Kawashiri SY, Shimizu T, et al. Assessment of both articular synovitis and tenosynovitis by ultrasound is useful for evaluations of hand dysfunction in early rheumatoid arthritis patients. *Mod Rheumatol* 2017;27:605-608.

16 Ten Brinck RM, van Steenbergen HW, Mangnus L, et al. Functional limitations in the phase of clinically suspect arthralgia are as serious as in early clinical arthritis; a longitudinal study. *RMD Open* 2017;3:e000419.

17 Burgers LE, Nieuwenhuis WP, van Steenbergen HW, et al. Magnetic resonance imaging-detected inflammation is associated with functional disability in early arthritis-results of a cross-sectional study. *Rheumatology (Oxford)* 2016;55:2167-2175.

18 Glinatsi D, Baker JF, Hetland ML, et al. Magnetic resonance imaging assessed inflammation in the wrist is associated with patient-reported physical impairment, global assessment of disease activity and pain in early rheumatoid arthritis: Longitudinal results from two randomised controlled trials. *Ann Rheum Dis* 2017;76:1707-1715.
